# Clinical characteristics, laboratory findings, and tolerance acquisition in infants with cow's milk protein allergy in a private center in Lima, Peru for the period 2021–2022

**DOI:** 10.1002/iid3.1246

**Published:** 2024-04-26

**Authors:** César Galván Calle, Cecilia Díaz‐Vásquez, Wilmer Córdova‐Calderón, Juan Gómez de la Torre, Edgar Matos‐Benavides, Crhistian Toribio‐Dionicio

**Affiliations:** ^1^ Asthma Allergy and Immunology Instituto Nacional de Salud del Niño – Breña Breña Lima Peru; ^2^ Instituto latinoamericano de Alergias Asma e Inmunologia (ILAAI) Lince Lima Peru; ^3^ Emedic Salud San Isidro Lima Peru; ^4^ Pediatrics Unit Instituto Nacional de Salud del Niño – San Borja San Borja Lima Peru; ^5^ Clinic Laboratorio ROE Surco Lima Peru; ^6^ New York Medical College, Metropolitan Hospital NY USA

**Keywords:** allergy, animals, autoimmunity, diseases, human, processes

## Abstract

**Background:**

Cow's milk protein allergy (CMPA) remains relatively understudied in Latin America.

**Methods:**

In this observational study, we enrolled 64 patients with a median age of 3 months, of whom 60% were male. Patients included had a history of IgE‐mediated reactions with IgE sensitization or non‐IgE‐mediated reactions or symptoms following exposure to cow's milk. They underwent skin prick test, ImmunoCAP, fecal calprotectin (FC), and fecal eosinophil‐derived neurotoxin (EDN), in addition to double‐blinded placebo‐controlled oral food challenges (DBPCFC), with clinical evolution and tolerance acquisition observed over 1 year.

**Results:**

Malnutrition was present in 78.1% of patients, and 87.5% had a family history of atopy, with 51.6% receiving exclusive breastfeeding. Gastrointestinal manifestations were prevalent in 90.6% of patients, followed by dermatological manifestations (10.9%), with only 2 experiencing anaphylaxis. IgE‐mediated CMPA was observed in only six patients. In those with non‐IgE‐mediated CMPA, FC had a median of 284 mg/dL (IQR: 138.5–415.5), while EDN had a median of 508.5 mg/dL (IQR: 160.25–868). One year after diagnosis, median FC significantly decreased (*p* < 0.0001), and malnutrition prevalence reduced to 17.1%. Moreover, 81% of patients acquired tolerance following DBPCFC, with 52% utilizing nutritional replacement formulas at diagnosis. Notably, 94% of those extensively hydrolyzed casein‐based formulas achieved tolerance (*p* = 0.08).

**Conclusion:**

Our findings provide a foundational framework for future investigations into CMPA diagnosis, tolerance acquisition, and the utilization of hypoallergenic formulas tailored to the unique characteristics of our region.

## 1 INTRODUCTION

Food allergies (FA) represent a growing healthcare problem globally, including in developing countries, likely attributable to the adoption of Westernized lifestyles in these regions.^1^ Additionally, epidemiological data on FA in these regions is limited due to low public awareness, inadequate physician education and training, limited access to healthcare and diagnostic tools, shortage of allergists, and overlap with other conditions such as malnutrition and childhood illnesses.^1^, ^2^, ^3^ It is important to mention that overdiagnosis is also reported; for example, in Peru, 40.9% of parents reported FA in their children, as documented in a national reference center for allergic diseases.^4^

Cow's milk protein allergy (CMPA) is the most frequently diagnosed FA among children younger than 1 year. However, precise data in developing countries remains elusive, as several studies rely on self‐reports, often lacking comprehensive assessments including family history, clinical characteristics, relationship with food exposure, utilization of FA tests, or conducting a double‐blinded placebo‐control food challenge (DBPCFC).^5^ Likewise, although using fecal calprotectin (FC) and eosinophil‐derived neurotoxin (EDN) in evaluating intestinal inflammation and diagnosing non‐IgE mediated CMPA is promising, their utilization remains limited, with studies showing variable results.^6^, ^7^

Therefore, our study aims to describe the clinical features, laboratory findings, and outcomes of oral challenges and tolerance acquisition in Peruvian infants diagnosed with CMPA younger than 1‐year‐old.

## 2 METHODOLOGY

This single‐center observational study was conducted in infants (<1 year) diagnosed with CMPA who attended a private allergic diseases center in Lima, Peru, in 2021. The study protocol received approval from the Ethics Committee of the Instituto Nacional de Salud del Niño, Breña, Peru.

### 2.1 Patient recruitment

Inclusion criteria encompassed infants with a history of an immediate allergic reaction (<2 h) following cow's milk or cow's milk‐containing product exposure, with evidence of IgE sensitization (specific IgE ≥5 kUA/L or a wheal diameter ≥6 mm in a skin prick test)^8^, ^9^ or an immediate allergic reaction during a DBPCFC for IgE‐mediated CMPA. Non‐IgE‐mediated CMPA cases involved infants with a history of non‐IgE manifestations after cow's milk or cow's milk containing‐products exposure or non‐IgE manifestations after DBPCFC (based on previous protocols)^10^, ^11^ with no evidence of IgE sensitization.

Allergen skin testing was performed using the epicutaneous technique with standardized cow's milk, egg white, egg yolk, peanuts, wheat, soy, fish, and shrimp extract solutions (Immunotek), while specific IgE levels were measured using ImmunoCap (Thermo Fisher).

Patients with genetic disorders or inborn errors of immunity or metabolism were excluded.

### 2.2 Data collection

Demographic characteristics, family history of atopy, clinical manifestations, and accidental food exposure data were collected. Patients with non‐IgE‐mediated CMPA underwent FC and EDN testing.

### 2.3 Tolerance monitoring

Following 1 year from diagnosis, patients underwent DBPCFC and were observed for 1 week to assess cow's milk tolerance. We described the hypoallergenic formula utilized and documented any adverse events observed during the 1‐year follow‐up.

### 2.4 Statistical analysis

With an anticipated CMPA prevalence of 3.5%, an acceptable margin of error of 10%, and a confidence level of 90%, we calculated a sample size of 64 patients using Epi Info software. The Mann–Whitney *U* test was employed to explore the relationship between symptoms and FC and EDN values.

## 3 RESULTS

A total of 64 patients, with a median age of 3 months (IQR: 1–5 months), were included in the study, with approximately 60% being male. Malnutrition was observed in 50 patients (78.1%), and C‐section delivery occurred in 68.7% of cases. Before CMPA diagnosis, 33 patients (51.6%) were exclusively breastfed. Additionally, 56 patients (87.5%) had a family history of atopy, with 39% reporting a family history of asthma and 71.9% indicating a family history of allergic rhinitis. Further demographic data is presented in Table 1.

**Table 1:** Demographic characteristics and history of 64 patients with CMPA.

Category	*n*	%
Age in months (IQR)	3	(1–5)
Sex		
Female	25	39
Male	39	60.9
Origin		
Lima	61	95.3
Provinces	3	4.7
Nutritional status		
Malnutrition	50	78.1
No malnutrition	14	21.9
Type of delivery		
Vaginal	20	31.3
C‐section	44	68.8
Delivery complications		
Cardiac arrhythmia	1	1.6
COVID‐19	1	1.6
Dystocia	2	3.1
Nuchal cord	1	1.6
Spontaneous hemorrhage	1	1.6
Neonatal jaundice	1	1.6
Oligohydramnios	1	1.6
Amniotic fluid leak	1	1.6
Placenta previa	1	1.6
Breech delivery	2	3.3
Pre‐eclampsia	4	6.3
Prematurity	1	1.6
Premature rupture of membranes	1	1.6
Fetal distress	1	1.6
Tachycardia	1	1.6
Feeding		
Exclusive breastfeeding	33	51.6
Mixed feeding	31	48.4
Family history		
Atopy	56	87.5
Asthma	25	39.1
Rhinitis	46	71.9
Eczema	4	6.3
Food allergy	9	14.1
Drug allergy	4	6.3

### 3.1 Clinical manifestations

The clinical manifestations of patients with CMPA are summarized in Table 2. Gastrointestinal manifestations were predominant, with 58 patients exhibiting symptoms such as colic (45.3%), diarrhea (43.8%), and regurgitation (25%). Dermatologic manifestations were observed in seven patients (10.9%), while anaphylaxis was only reported in two cases. Most patients were diagnosed with non‐IgE‐mediated CMPA; only six patients (9.3%) had IgE‐mediated CMPA.

**Table 2:** Clinical manifestations of 64 patients with CMPA.

Category	*n*	%
Dermatological manifestation		
Erythema	5	7.8
Wheal	4	6.3
Angioedema	2	3.1
Eczema	2	3.1
At least one	7	10.9
Respiratory manifestation	2	3.1
Gastrointestinal manifestation		
Vomiting	1	1.6
Diarrhea	28	43.8
Proctocolitis	13	20.3
Regurgitation	16	25.0
Colic	29	45.3
At least one	58	90.6
Anaphylaxis	2	3.1

### 3.2 IgE sensitization

Among 13 patients with positive skin prick tests, 5 were reactive to cow's milk, 5 to egg white, 1 to cow's milk and egg white, 1 to fish, and 1 to peanuts. Of the six patients with a positive skin prick test for cow's milk, all presented specific IgE levels >5 kUA/L by ImmunoCAP.

### 3.3 Fecal biomarkers

Regarding fecal biomarkers, the median levels of EDN and FC were 508.5 mg/dL (IQR: 160.25–868 mg/dL) and 284 mg/dL (IQR: 138.5–415.5 mg/dL), respectively. Significant associations were observed between FC levels and regurgitation or vomiting (*p* = 0.017), as well as between EDN levels and regurgitation or vomiting lasting less than 14 days (*p* = 0.017). Further details are provided in Table 3.

**Table 3:** Fecal biomarkers findings of 64 patients with CMPA.

Test	Median (IQR)	Symptom	*p* (present vs. absent)
Fecal neurotoxin	508.5 mg/dL (160.25–868)	Proctocolitis	0.534
		Diarrhea	0.801
		Proctocolitis or diarrhea	0.547
		Regurgitation	0.773
		Vomiting	1
		Regurgitation or vomiting	0.131
		Colic	0.616
		Anaphylaxis	0.472
Fecal calprotectin	284 mg/dL (138.5–415.5)	Proctocolitis	0.534
		Diarrhea	0.450
		Proctocolitis or diarrhea	0.664
		Regurgitation	0.773
		Vomiting	1
		Regurgitation or vomiting	**0.014**
		Colic	0.315
		Anaphylaxis	0.472

*Note*: Bold value is statistically significance.

### 3.4 Tolerance acquisition

Among the study cohort, six infants met diagnostic criteria for IgE‐mediated CMPA based on clinical history and laboratory findings and did not undergo DBPCFCs. The remaining 58 patients were considered possible non‐IgE‐mediated CMPA, prompting the performance of DBPCFC in all cases. This resulted in a total of 67 challenges, with 86% yielding positive results. At the time of CMPA diagnosis, 51 patients were using nutritional replacement formulas, among which 37.5% used extensively hydrolyzed casein‐based formulas, 33.3% used partially hydrolyzed rice‐based formulas, 20.9% used amino acid based‐formulas, and only 4 patients used soy‐based formulas.

After 1 year of follow‐up, the median FC level was 104 mg/dL (IQR: 72–136 mg/dL), whereas the median EDN level was 620 mg/dL (IQR: 406–1316 mg/dL). A significant difference was observed in FC levels 1‐year postdiagnosis (*p* < 0.0001), while no significant difference was found with EDN levels.

Eighty‐one percent of patients (*n* = 52) acquired tolerance to cow's milk after 1 year of follow‐up based on DBPCFC results. Thirthy‐three patients used nutritional replacement formulas at least three times a week during the 1‐year follow‐up period, with 16 patients using extensively hydrolyzed casein‐based formula, of whom 15 (94%) acquired tolerance to cow's milk after 1 year.

Seventeen patients used other formulas, among which 71% acquired tolerance (*p* = 0.08). Notably, patients using an extensively hydrolyzed casein‐based formula exhibited a median FC reduction to 89 mg/dL (IQR: 65–112) after 1 year (*p* = 0.12). Tolerance rates after 1 year were as follows: soy‐based formula (50%), amino acid‐based formula (66%), and partially hydrolyzed rice‐based formula (78%). None of the patients utilizing nutritional replacement formulas experienced allergic or severe adverse events associated with formula consumption. Malnutrition decreased to 17.1% after 1 year of using formula.

## 4 DISCUSSION

Our study describes infants under 1 year of age diagnosed with CMPA through stringent criteria. These infants were predominantly delivered via C‐section, skewed toward males, with a notable proportion experiencing malnutrition at diagnosis. Additionally, they exhibited familial allergic respiratory diseases and a comparable distribution concerning diet type at diagnosis. This clinical profile is similar to other South American regions, as reported by Errazuriz et al., highlighting a predominantly male population with familial atopy history and a similar diet distribution, albeit with a healthier status in their cohort.^12^ Similar observations were made in a prospective study conducted in a private center in Chile.^13^ However, in regions such as France, Kalach et al. reported similar clinical patterns but with a lower proportion of exclusive breastfeeding at diagnosis (33%, less than that in our study).^14^ Exclusive breastfeeding, slightly more prevalent in our CMPA patients' diagnosis (51.66% vs. 48.44%), has been shown to exert a protective effect against CMPA development compared with early infant formula use, as evidenced by a systematic review.^15^

The majority of infants in our study were diagnosed with CMPA within the first 5 months of life, primarily presenting with gastrointestinal symptoms, notably colic and diarrhea. These findings align with reports from other settings.^12^, ^14^ Moreover, several gastrointestinal manifestations observed in CMPA clinical presentations, such as regurgitation, colic, or constipation, may resemble functional conditions common in healthy infants. Hence, a focused clinical history and examination are crucial for distinguishing between physiological and pathological conditions such as CMPA. While all patients in our study with gastrointestinal symptoms underwent DBPCFC, facilitating confirmation of diagnosis may not always be feasible in every clinical scenario, underscoring the reliance on medical history for accurate diagnosis.^16^

Our study evaluated nutritional status at diagnosis, revealing a significant proportion (35.93%) with varying degrees of malnutrition, reflecting challenges posed by public health systems, poverty, and insufficient educational strategies in Latin American countries. Remarkably, 1 year after CMPA diagnosis, the prevalence of malnutrition considerably decreased to 17.18%, emphasizing the pivotal role of early CMPA detection.^17^

Exploration of IgE‐mediated responses revealed six infants testing positive for cow's milk among the 64 infants (9%). Two of these six infants presented with anaphylaxis, characterized by predominant skin and respiratory involvement (hives and bronchospasm). Thus, the majority of our CMPA patients (91%) exhibited a non‐IgE‐mediated type, consistent with findings reported in multiple publications.^18^, ^19^ Studies in Latin America reporting IgE‐mediated versus non‐IgE‐mediated CMPA frequency display variability. Arancibia et al. reported exclusively non‐IgE‐CMPA cases in their cohort (20 patients) using food challenges,^13^ whereas Mehaudy et al. documented a 27.6% frequency of IgE‐mediated CMPA in Argentina.^20^ Discrepancies may stem from differences in age at diagnosis, diagnosing specialists, patient demographics, and socioeconomic status. In our study, most patients were diagnosed before 5 months of age, referred by allergists, and hailed from socioeconomic backgrounds above the population average.

Few studies in Latin America have utilized DBPCFC for CMPA diagnosis. Errazuriz et al. conducted DBPCFC in 14% of their patients,^12^ whereas Arancibia et al. performed it in all their patients, albeit employing open oral challenges.^13^ Goncalves et al. conducted single‐blinded oral challenges in Brazil.^21^ In our study, DBPCFC was performed in 91% of patients and in all suspected non‐IgE‐mediated CMPA cases, yielding an 86% positivity rate. DBPCFC positivity rates vary widely in the medical literature,^22^, ^23^ likely influenced by factors such as duration of prior elimination diet, age at diagnosis, predominant CMPA symptomatology, and clinician's initial suspicion accuracy, among others.

The use of fecal biomarkers in diagnosing non‐IgE‐mediated CMPA is an ongoing area of research. Trillo et al. reported that an FC level below 138 μg/g could exclude non‐IgE‐mediated CMPA, suggesting its utility for prospective follow‐up at 1‐ and 3‐months postdiagnosis.^24^ Similarly, Rycyk et al. found FC (88.9%) and EDN (84%) prevalence in 27 patients with non‐IgE‐mediated CMPA.^6^ However, Roca et al. did not find significant differences in FC or EDN levels among 30 patients with non‐IgE–mediated CMPA, though not all patients underwent oral challenges.^7^ Our study evaluated all non‐IgE–mediated CMPA patients for FC and EDN, revealing significant FC differences in cases presenting with regurgitation/vomiting. Significant EDN differences were also noted in patients experiencing recent symptoms (within 2 weeks) of diarrhea and regurgitation/vomiting. Furthermore, a significant median reduction in FC levels was observed after 1 year of observation. Larger‐scale studies with specific designs are warranted to validate our findings.

Tolerance acquisition to cow's milk 1‐year postdiagnosis mirrored findings from other regions.^12^ In our analysis of patients utilizing nutritional replacement formulas, those using extensively hydrolyzed casein‐based formulas exhibited greater tolerance acquisition (94%) and FC level reduction compared with those using other formulas (71%), though statistical significance was not reached (*p* values of 0.08 and 0.12, respectively). This trend, identified in our study, has been supported by previous clinical trials.^25^

The strengths of our study include its comprehensive evaluation of infants under 1 year of age diagnosed with CMPA, including utilization of DBPCFC in all patients with non‐IgE‐mediated CMPA. Furthermore, our study contributes novel insights by exploring fecal biomarker levels in non‐IgE‐mediated CMPA, filling a crucial gap in the literature regarding diagnostic approaches in this subset of patients. Additionally, our study provides valuable data on nutritional status and tolerance acquisition, shedding light on the effectiveness of different formula types in managing CMPA. However, our study also possesses some limitations. Being a single‐center observational study, our findings may not be generalizable to the broader population and the small sample size analyzed. In addition, our study explores fecal biomarkers in non‐IgE‐mediated CMPA, the interpretation of these biomarkers remains subject to ongoing research and validation, highlighting the need for further investigation in larger, prospective studies.

In conclusion, our study is among the few in Latin America that elucidates the clinical characteristics, laboratory tests, DBPCFC, and tolerance outcomes in infants under 1 year of age with CMPA. Furthermore, it is likely the first in the region to explore fecal biomarker levels in non‐IgE‐mediated CMPA diagnosed with DBPCFC. Our findings provide a foundation for future research investigating CMPA diagnosis and tolerance acquisition, considering the unique challenges faced in our region.

## AUTHOR CONTRIBUTIONS

**César Galván Calle**: Conceptualization; data curation; formal analysis; funding acquisition; investigation; methodology; project administration; resources; software; supervision; validation; visualization; writing—original draft; writing—review and editing. **Cecilia Díaz‐Vásquez**: Data curation; formal analysis; writing—original draft; writing—review and editing. **Wilmer Córdova‐Calderón**: Data curation; formal analysis; writing—original draft; writing—review and editing. **Juan Gómez de la Torre**: Data curation; formal analysis; writing—original draft; writing—review and editing. **Edgar Matos‐Benavides**: Data curation; formal analysis; writing—original draft; writing—review and editing. All authors contributed equally to the research process, data analysis and drafting of the article.
